# 

**Published:** 1909-03

**Authors:** 


Lydston on Glass Houses. Ajax Defies the Lightning.

Texas Castration Bill.

Vol. XXIV. MARCH, 1909. No. 9.

### TEXAS MEDICAL JOURNAL

Indexed
S. G. O.

"RED BACK"

PUBLISHED AT AUSTIN, TEXAS, BY

F. E. DANIEL, M. D., - - Proprietor

PRINTED BY THE VON BOECKMANN-JONES CO.

Subscription, $1.00 a Year, in Advance. - - - Single Copies, 10 Cents

Entered at the Postoffice at Austin, Texas, as Second-class Mail Matter

